# Enhancing multi-sectoral collaboration in health: the open arena for public health as a model for bridging the knowledge-translation gap

**DOI:** 10.3389/frhs.2023.1216234

**Published:** 2023-09-18

**Authors:** Christian Pradier, Marta A. Balinska, Laurent Bailly

**Affiliations:** ^1^ Department of Public Health, Nice University Hospital, University of Côte D'Azur, Nice, France; ^2^ Clinical Research Unit (UR2CA), Nice University Hospital, University of Côte D'Azur, Nice, France

**Keywords:** policy dialogue, multi-sectoral collaboration, community-Based participatory research, health promotion, knowledge sharing

## Abstract

Effective public health interventions at local level must involve communities and stakeholders beyond the health services spectrum. A dedicated venue for structured discussion will ensure ongoing multi-sectoral collaboration more effectively than convening *ad hoc* meetings. Such a venue can be created using existing resources, at minimal extra cost. The University Hospital in Nice (France) has established an Open Arena for Public Health which can serve as a model for promoting collaborative partnerships at local level. The Arena has been successful in implementing sustainable interventions thanks to a set of principles, including: non-hierarchical governance and operating, fair representation of stakeholders, consensus as to best available evidence internationally and locally, policy dialogues: open, free-flowing discussions without preconceived solutions, and an experimental approach to interventions.

## 1. Introduction

As we move towards the second quarter of the 21st century, evidence-based medicine has taken the lead on expert clinical practice. Likewise, we are increasingly moving towards evidence-informed health policies and away from interventions guided by expertise or political will only. In parallel to the emergence of evidence to support decision-making in medicine and public health, experience is demonstrating the key role of involving communities in the design of public health interventions (1–3). Collaborative governance and community-based participatory research have made remarkable strides since the turn of the century. Increasingly, funders are requiring not only community participation in health promotion research (4), but cultural competency of relevant stakeholders and officials (5).

Stakeholder involvement in the design of interventions is necessary not only to determine their characteristics and scope, but also their uptake and ultimately their sustainability, without which even the most carefully designed initiatives will not produce the desired effects (6). Historically, many public health campaigns have been based on informing the public on the assumption that knowledge alone is enough to change behavior. But now we know that communities must also have the means to change their behavior.

The World Health Organization is increasingly promoting policy dialogues as the key knowledge translation tool for evidence-informed policy making (7). “Policy dialogues”—while not yet benefiting from an official definition—are broadly described as an interactive knowledge-sharing mechanism among a comprehensive range of stakeholders. Their use is encouraged for response to major public health problems, especially those which “resist solution, where there is no clear “right” answer and a number of different interests, priorities and values are in tension” (8). For instance, the WHO recently called for policy dialogues to tackle the obesity epidemic across the European region (9).

For the past 15 years, the Open Arena for Public Health (Espace Partagé de Santé Publique) in the department of Alpes Maritimes (South Eastern France) has been bringing together academics, decision-makers, and community representatives on a regular basis to tackle local health challenges. Although not formally designated as such, the de facto mechanism of concertation has been policy dialogues. Elsewhere, policy dialogues have been convened mostly on an *ad hoc* basis (10) (11, 12), but there is growing recognition that their systematic use—as in Alpes Maritimes—would be beneficial to promoting regular interaction of stakeholders in a given community setting (13, 14).

In this policy brief, we make recommendations for enhancing multi-sectorial collaboration via a dedicated space such as the Open Arena for Public Health in Nice which engages in ongoing policy dialogues.

## 2. Functioning of the open arena for public health

### 2.1. The open arena and the public health landscape in France

France has a long history of centralization. The country is divided administratively into 5 regions and 101 departments (including overseas). Piloting and coordination of health policies are ensured at the national level and translated locally via regional health agencies.

Any public health/health promotion intervention which does not fall directly within the prerogative of the department or the municipality (such as school lunches or sports venues) must be approved for funding by the regional health agency. Broadly speaking, the public health landscape remains rigid and top heavy with limited scope for adapting national policies to local contexts.

To overcome this rigidity, the University Hospital of Nice instituted an Open Arena for Public Health to be managed by the hospital's Department of Public Health. The aim of the Open Arena has been to improve the health status of communities living in Alpes Maritimes through collaborative partnerships among community representatives, civil society organizations, local health stakeholders, and academic institutions. From its inception, it has sought to federate across parties and respond in a timely fashion to evolving health determinants and population expectations, in line with the principles of health promotion and the new public health. The Arena was established using existing resources—i.e., the time and expertise of staff within the Department of Public Health—thus incurring no extra cost.

The Arena operates via a Steering Committee and an Operational Board. Project selection and decision-making are based on public health data, academic expertise, and community participation using a policy dialogue mechanism. The Open Arena also carries out consultative and technical support activities. As such, it breaks down administrative barriers among existing institutions and fosters collective thinking and collaboration among professionals and community members unused to working together. Community representatives are identified by a snowballing process, starting with civil society organizations and local stakeholders known to the municipality or greater Nice area. All partners volunteer their time to ensure cost containment. When funding for a specific project is required, it will be sought within the budgets of partnering institutions.

The Open Arena will meet whenever a complex public health priority is identified, such as poor uptake of cancer screening (15), or medical desertification in rural areas. As a department of France suffering from marked social disparities, the Arena is particularly concerned with health problems linked to inequalities and inequities. The process for convening the Arena can be reactive, e.g., in response to community concerns over a health-related issue such as pollution, or proactive as when the Steering Committee alerts members to a major health concern such as high prevalence of pediatric obesity in certain Nice neighborhoods. Discussion is rooted in scientific evidence provided by the Department of Public Health, but evolves freely as participants share their knowledge, experience, and skills. Typically, this encourages thinking out of the box and leads to innovative proposals involving new partnerships.

### 2.2. Example of an open arena intervention: preserving autonomy for the elderly

One of the first requests made to the Arena's Steering Committee was to think about new housing solutions for the dependent elderly. Several policy dialogues were convened, involving a wide range of stakeholders. The discussions shifted their focus from housing to the preservation of autonomy in senior citizens and came up with a comprehensive model for reducing loss of autonomy in the elderly. In line with the Ottawa Charter for Health Promotion, the Open Arena wished to empower older individuals with regard to their health through enhanced physical activity and ongoing social interaction. Over the past decades, several community-based health interventions have been developed to promote healthy ageing, specifically through physical activity (16–18).

The pilot intervention (the 4-S initiative: Saint-Roch—Sport—Solidarity—Senior Citizens) consisted of improving the urban environment of a socially disadvantaged neighborhood of Nice (Box 1). Consultations were carried out with local senior citizens, thus leading to a walking route in line with the expectations of those who would use them (19). The walking routes were also a means of strengthening social ties through meeting places such as open areas and shops. The evaluation of the intervention indicated enhanced quality of life for older individuals through a holistic approach including physical, social and mental well-being (20). Importantly, the intervention fit within the model of integrating progressive loss of autonomy into the life course of older individuals. This model seeks to create an environment conducive to “better ageing”, including a network of medical and social support, and suitable housing for those who become too dependent to live in their own home. Thus, institutionalized living is no longer the prime issue to be addressed, but emerges as a solution for the most vulnerable. Further, housing for the dependent elderly is foreseen within the neighborhood where they have previously lived.

Box 1 **The 4-S initiative: Saint-Roch, sport, solidarity & senior citizens.**

**Table:** 

**Problem:** lack of housing for the dependent elderly.
**Shared Vision:** maintaining autonomy in senior citizens is a priority.
**Context:** preventing institutionalization is humanly preferable and less costly for the state than increasing the number of beds for the elderly.
**Evidence base:** enhancing senior citizens' regular physical activity can prevent loss of autonomy and improve quality of life in older population groups.
**Solution identified:** encourage neighborhood walking as a freely accessible means of physical exercise for all senior citizens.
**Pilot community:** the Saint-Roch district, a low-income neighborhood in Nice lacking adequate sidewalks and green spaces, with poorly controlled traffic.
**Main intervention:** urban walking trails.
**Result:** improvement in endurance score for Saint-Roch residents compared to residents of a neighborhood without urban walking trails.
**Outcome:** urban walking trails introduced in four other Nice neighborhoods.

Multi-sectoral partnering such as the Open Arena for Public Health is unique in France, yet can be replicated in almost any setting. In order for it to achieve its purpose, a number of actions can be recommended.

## 3. Actionable recommendations

### 3.1. Strong but non-hierarchical governance

The Open Arena for Public Health does not have a specific legal structure or dedicated funding. It operates through a Steering Committee, an Operating Board, and Project Groups. It is operational and flexible, and encourages both a bottom-up and top-down approach, although the ultimate decision-making power remains with the Steering Committee. Members volunteer to join working groups and dedicate their working time, thus allowing the Arena to function without incurring extra cost. In face of a specific challenge or problem, leaders will articulate a vision which can be shared by all, but they do not plan any interventions beforehand or present preconceived ideas. Instead, policy dialogue and interaction allows stakeholders to come up with original proposals and solutions in line with the shared vision.

Specifically:

• The Steering Committee is the strategic core. It includes all decision-makers responsible for identifying partners, funding sources, and communication strategies. The Steering Committee maintains trust and cooperation among stakeholders and creates an environment favorable to change over time. It meets once a year.
• The Operating Board, made up of stakeholders and academics, meets at least three times a year. It develops the strategies required for change to materialize. Each time a new project is launched, a dedicated team is set up and evaluation protocols are developed. The Operating Board is responsible for coordinating these teams and making recommendations to the Steering Committee on the basis of collective discussions.
• The Project Groups bring together stakeholders (often technical experts) directly involved in implementing interventions. They are in charge of representing communities’ needs and developing approaches which allow individuals to be actors of their own health. The Project Groups also identify and report any problems encountered in the field and suggest solutions. They meet at different times depending on how the intervention is progressing and which actions need to be taken.

This three-tiered structure is intended to be both adaptive and self-organizing. Participants have freedom of action and influence each other collectively. Such room for maneuver, sharing of experience, and pooling of skills leads to creative experimentation in responding to local health challenges. Participants in Open Arena discussions must feel they are on equal footing when analyzing evidence and seeking solutions. As observed in the “Model of Research-Community Partnership”, described by Brookman-Frazee, facilitating factors for collaborative processes depend on non-hierarchical, collegial relations among partners based on mutual respect and trust (21).

### 3.2. Fair representation of stakeholders

In face of a given challenge, it is essential that the entire range of stakeholders be represented, and contribute to the policy dialogue. Failure to invite a key stakeholder can compromise the identification of a workable solution and/or its uptake in the community.

Bringing together representatives from different organizations, communities, disciplines, backgrounds, and cultures to exchange knowledge, discuss evidence, and suggest ways forwards always presents challenges. There must be mutual respect and acceptance that consensus cannot always be secured as a single set of actions, but will often take the form of a multiplicity of perceived solutions (a hallmark of policy dialogues).

For instance, regarding the model of loss of autonomy in the elderly and the 4-S initiative, academic and public health professionals contributed their knowledge and expertise; community representatives provided citizen's feedback regarding their specific social, environmental, and cultural context; and municipal policymakers were able to discuss financing in the context of competing priorities so as to make the best use of public funds.

### 3.3. A shared vision, but not preconceived solutions

While the policy dialogue itself remains an open and free-flowing discussion with minimal rules, it is essential that the participants start out with a shared vision of the problem at hand, and the importance for public health of addressing it adequately.

Prior to elaborating the walking trails intervention in a disadvantaged Nice neighborhood, all participants agreed that solutions needed to be found in face of lack of housing for the dependent elderly and, generally speaking, that more needed to be done to promote healthy ageing in the city of Nice. They were thus fully engaged in the need for and process of change.

### 3.4. Thinking global—acting local

The Open Arena's initiatives are aligned with major international objectives such as reducing obesity or creating healthy cities, but conceived on a very local scale—most often in terms of neighborhoods. Beyond considerations of experimentation and tailored interventions, the neighborhood is the nexus of everyday life in which individual and collective responsibility take on concrete meaning.

In the United States, the “Active Living by Design” national program was established to help 25 programs resulting from interdisciplinary collaborative partnerships create healthy urban environments and increase physical activity and social support within neighborhoods (22, 23). This kind of community action model has been successfully applied in other countries (24, 25) and served as an inspiration to the Open Arena in Nice when developing its own interventions to promote healthy ageing.

Taking a territorial approach (districts) means that population needs—both expressed and unexpressed—can be deliberately considered. Interventions at different levels of the health continuum contribute to promoting healthy lifestyles through educational and environmental strategies. These actions should be developed according to a life course approach, by intervening upstream on the determinants of health to prevent the loss of autonomy, and better meet the needs of an ageing population.

### 3.5. Discussions based on evidence

The golden rule for policy dialogues is that they be based on available evidence. This evidence should be summarized in plain language and shared with all participants before the initial meeting. Evidence is based on national and international data (Santé Publique France, WHO, scientific publications, etc.) but also draws on local aggregated data made available by the national income tax and statistics agencies. The strength and appropriateness of this evidence may be debated, but it serves as a starting block on which to build. Often, this evidence needs to be completed.

In what led up to the enhanced physical activity initiative, social science researchers introduced the life course approach to discussions around healthy ageing and relevant information was made available to all participants.

Interestingly, partners involved in the dialogues initially each had their own project in mind for enhancing physical exercise for the elderly. But the discussions led to a synergetic effect and identified urban walking trails as the best practicable solution.

Focus group discussions with neighborhood residents collected experiences of physical activity, requirements to improve walking opportunities, and proposals to overcome perceived difficulties. Participants clearly stated that heavy traffic, sidewalk parking, unavailable pedestrian passages or limited vision at crossings led to a sense of insecurity and discouraged them from walking in their own neighborhood. They then proposed their own itinerary which included congenial spots and avoided unpleasant ones. Such specific input was obviously crucial to creating urban trails which people would actually use.

### 3.6. Experimental approach

The beauty of policy dialogues is that they can, and often do, lead to new ideas (or old ideas which have been forgotten). All the health promotion interventions conducted by the Open Arena are first tested on a small scale (usually a pilot study within a target community) and replicated only if successful.

The Saint-Roch district was chosen as the target neighborhood for the 4-S Initiative being a low-income neighborhood in the city center. Another low-income neighborhood was selected as the control. The goal was to assess the combined impact of an organized urban walking circuit and individual coaching on female senior citizens’ physical well-being and quality of life. Older women in the target and control districts were randomly allocated to receive coaching. The invention was funded by the regional health agency and targeted over 4,000 citizens above the age of 64. At three months, the endurance score was higher in the improved urban environment group, whether coupled with coaching or not (20).

## 4. Conclusion

The Open Arena for Public Health is an example of a local initiative which has led to substantial social and political innovation in improving population health in Alpes Maritimes (Table 1). The Arena clearly arose within the premises of the new public health, meaning “community participation in health policy development and implementation of programs, [emphasis on] primary health care and health promotion, and inter-sectoral cooperation involving agencies whose influence impinges on health” (26). It seeks to bridge the gap between academics, on the one hand, and policy makers and implementers on the other, in order to improve community health.

**Table 1:** Examples of interventions resulting from policy dialogues within the open arena for public health.

Problem	Evidence/Vision	Stakeholders	Intervention/Recommendation	Outcome(s)
Neighborhood exposure to waste incinerator & public concern	Potential elevated cancer risk for neighborhood inhabitants	Métropole Nice CA, Alpes Maritimes Department, Regional Health Agency, Nice University Hospital, Neighborhood representatives	Surveillance in collaboration with civil society organizations	Cancer registry, geo localized statistics
Lack of adequate medical care for the elderly	The complexity of care requires integrated services	Alpes Maritimes Department, Regional Health Agency, Nice University Hospital, all local health networks, civil society organization representing the elderly	Integrated, coordinated care to help the elderly stay in their own home	“C3S”—Dedicated center for health and social support (Centre de soutien santé social)
Medical desertification in rural areas	Financial compensation does not suffice to motivate physicians	City of Nice, Métropole Nice CA, Alpes Maritimes Department, Nice University Hospital, all local health networks, GP representatives	Develop tailored marketing policies to enhance living conditions in rural areas	Improved access to healthcare in rural areas (expected).
Hospital bed saturation post COVID-19	Follow-up of hospitalized COVID-19 patients can be carried out at home	Regional Health Agency, Nice University Hospital, “C3S”, patient representatives	Dedicated human resource from “C3S” to speed up hospital discharge	Saturation problem resolved
High prevalence of obesity in young children in socially disadvantaged neighborhoods	A wide range of factors across a child's everyday life contributes to overweight and obesity	City of Nice, Métropole Nice CA, Alpes Maritimes Department, Nice University Hospital, Ministry of Education, Civil Society Organizations	360° approach to healthy lifestyle at neighborhood level (modelled on Amsterdam Healthy Weight Approach)	Improved BMI z-scores, healthier lifestyle habits at year 5 (expected)

That is how—when it was called upon to deal with the problem of lack of housing for the dependent elderly—it focused on means to improve senior citizens’ overall health status, thus allowing them to remain in their own homes for as long as possible. The actions decided upon involved a wide spectrum of stakeholders, well beyond the health system. Such diversification of stakeholders, skills, and expertise clearly enhances both tacit and explicit knowledge sharing, and also leads to varied interpretations and intermediary solutions arising from mutual exchange and learning. Within this context, academics have played a key role by framing discussions within the most recent concepts in public health.

Arguably the principle hurdle which the Arena has faced time and time again is maintaining the horizontal approach to problems in a country where the model of governance is overwhelmingly top-down. If constant efforts are not made to keep the balance among stakeholders and maintain fluid cross-over as well as top-down-bottom-up processes, initiatives will fall flat or only partially reach their objectives.

The COVID-19 pandemic has once more highlighted the impact of social inequities on health outcomes (27). Solutions to address the consequences of such inequities must be based on evidence and involve actors beyond the health system. Yet policy interventions based on evidence entail huge efforts to harness available knowledge, share it, overcome conflicting views and priorities, and translate it into action.

We believe that the Open Arena for Public Health can serve as a model for ensuring ongoing exchange and answers to complex health challenges at community level, allowing change and innovation to come about as a result of collective intelligence and ongoing policy dialogues.
